# Recipients’ experience with information provision for electroconvulsive therapy (ECT)

**DOI:** 10.1186/s12888-022-03720-w

**Published:** 2022-02-04

**Authors:** A. Coman

**Affiliations:** Centre for medical ethics, Institute for health and society, University of Oslo, P.O. Box 1130, Blindern, 03168 Oslo, Norway

## Abstract

**Background:**

Despite improvements, studies continue to report unsatisfactory provision of information before, during and after electroconvulsive treatment (ECT).

**Aims:**

The study explores participants’ experiences with information provision about ECT.

**Methods:**

In-depth interviews with 21 participants (21– 65 year-old) were conducted. Thematic analysis resulted in identification of four themes: pre-treatment knowledge, experience of informed consent, the need for information depth and life after ECT. The study includes user involvement.

**Results:**

Although some participants were satisfied with information provision, the majority experienced an education deficit throughout the treatment period. Their consent was based mostly on oral information, insufficient and unvaried information on official health websites and media. Further, patients reported a lack of follow-up services that can attend to (neuro) psychological concerns.

**Conclusions:**

Better access to updated factual and narrative information should support patient education and autonomy. Active use of diary writing, better follow-up and more varied representations of experience with ECT in media and health information sites are necessary to educate, improve consent processes and reduce stigma.

## Background

Electroconvulsive treatment (ECT) is argued to have high remission rates for individuals with affective disorders unresponsive to psychotherapeutic or psychopharmacological treatments, although the relapse rates are high after 6 months and require maintenance medication or additional ECT [1–3]. ECT has remained controversial and the public’s perception of ECT is largely negative, seeing it as an ‘icon of psychiatric abuse and a remnant of early medicine’ [4]. This controversy is maintained in part by a collective memory of science’s dark past and practice, and ECT’s continued negative portrayal in popular culture and the media [5]. Indeed, many doctors tout it as an unfairly stigmatized treatment that can offer powerful relief [4]. One issue at the centre of this debate concerns the strength of evidence, and whether earlier placebo studies can withstand the proof of evidence required in modern medicine. Supporters of ECT point to the need to see the wider evidence available, where modern randomized controlled trials available, although few and mostly comparing different ECT techniques, are complemented by evidence from observational and audit data, and decades of clinical experience [6].

Controversy also revolves around effects on cognition, of which memory is the most debated and researched. Substantial data show that ECT recipients’ neurocognitive functions return to baseline after a few days or weeks [7–10], or are even improved and ‘boosted’ [11]. Nevertheless, there are knowledge gaps on long-term effects; moreover, some studies find that in community samples, some individuals experience irreversible retrograde amnesia, unable to recall events, personal memories or information, extending to more than 6 months [11, 12]. Subjective memory complaints have often been opposed by objective neuropsychological studies [13–15], or explained by low mood, prior poor cognitive functioning, older age [16] or negative expectations [15]. However, scant knowledge beyond mean effects from small studies, and limitations of the current batteries of tests to measure aspects of subjective memory, require further research on the extent or nature of subjective memory complaints in patients who have received ECT [13].

Studies that have explored recipients’ experience with information have revealed that although some patients experience ECT as beneficial, many still experience insufficient information about its adverse effects [17–19]. Some of these studies were consumer-led studies, which tend to differ from clinician-led studies that report more satisfied and informed patients [19]. The negative reports may be explained by patients’ increased honesty with non-clinicians, but also older ECT techniques that might have resulted in greater cognitive deficiencies, and less knowledge on the extent of those cognitive deficiencies. Nonetheless, even recent studies continue to report the need for patients and families to be better informed about ECT [20–23].

Information is especially relevant for a treatment that is polarized and where evidence is so heterogeneous that drawing meaningful conclusions is challenging [24]. The ethical principle of respect for autonomy requires that a person makes decisions voluntarily, holds decisional capacity and has been provided with sufficient information [25]. However, participants’ mental condition [26], the different information needs of individuals [27], and clinicians’ downplaying of adverse effects and criticism [28] further complicate the issue of valid and informed consent [26].

Higher patient satisfaction with information and treatment has been reported as a result of accreditation services’ work (UK) to improve information, and include patient experiences in the accreditation process [12, 29]. However, the Norwegian guidelines for ECT (2017) do not detail what constitutes sufficient information and how to ensure valid consent, leaving it up to each treating institution to decide what information to provide. In this diverging informational landscape, the patient—often in a vulnerable situation—may find it daunting to navigate the different perspectives. There are no studies, to the authors’ knowledge, that report quality improvement work or patient experiences with treatment and information provision in the Norwegian context. This article aims therefore to fill a gap and explore participants’ experience with information provision during and after ECT.

## Methods

### Recruitment and participants

An advertisement describing the study was posted on the Institute for Health and society’s website and on the website of the most prominent national mental health users’ organization. Sixteen participants contacted the researcher. The researcher also presented the study to a treatment team in three psychiatry departments in one of Norway’s largest cities. Seven individuals contacted the researcher or granted permission to be contacted via phone; two participants later decided not to participate. In total, 21 participants (21–65 years of age at the time of the interview) consented to participate in the study. Most interviews took place at the university facilities and were conducted between June 2017 and October 2018. Interviews lasted between 45 to 90 min, were audio recorded and transcribed verbatim. The aim was to interview participants three to six months after the last ECT in order to exclude any transitory memory problems. We did not have access to the medical records so the number of ECT and information received is self-reported. None of the participants were hospitalized or undergoing ECT at the time of the interview. All participants were White, ethnic Norwegians from different regions of the country. Demographic characteristic are presented in Table 1.

**Table 1:** Demographic characteristics of participants

Characteristic (n)	
Gender	
Male	3
Female	18
Age range (at time of ECT)	18–65
18–19	3
20–30	7
30–40	7
40–50	2
50–60	0
60+	2
Diagnosis	
Unipolar depression	12
Postpartum depression	3
Bipolar disorder	6
Time of treatment	
2000–2010	8
2010–2018	13
Number of courses	
One course	12
Multiple courses	6
Unsure	3
Time since last course	
Within 6 months	3

### User involvement

This project involved a reference group comprised of two researchers (.A.C. and a physician/ethicist), a physician, two earlier recipient of ECT and one family member. This group discussed the interview guide. The reference group was not involved in the analysis but after the researcher performed the analysis, she presented the findings to the user representatives. This is one of few studies where individuals with prior ECT experience were directly involved in the interviews (13). Participants gave their written consent before the interview. The interview guide contained questions about participants’ experience with procedures for ECT, information, effects, care and perception of causes and treatment for their depression. The principal investigator (AC) conducted all interviews and the user representative followed-up with questions when relevant. The user representative did not talk about her personal story or opinions on ECT in order to avoid influencing participants’ responses. The guide was employed to safeguard any departure from the intended topics and to maintain research integrity [30].

### Ethics statement

The project fell within the remit of health research and was approved by the Regional Ethics Committee South-East Norway (2017/2208). Participants signed a written consent form. They were not paid for their participation but their travel expenses were reimbursed. Participants were informed that they could talk to a clinical psychologist affiliated with the research institute if they experienced emotional distress after the interview; none of the participants used this service. Data were anonymized and stored via the university’s data protection system.

### Data analysis

Data were analysed in a thematic analytic manner using NVivo software [31]. After reading each interview and making notes, the author identified codes (meaning units) related to information in the interview sections. In order to ensure trustworthiness, two other researchers read and analysed three interviews, and the coding scheme was adjusted accordingly [32, 33]. The codes were then grouped into higher-order nodes that reflected the relationship between codes within and across the interviews. Information was seen not only in relation to consent but also in a broader sense, related to the participants’ experience of ECT in hospital and after they had returned home following treatment.

## Results

The following themes were identified:

### Pre-treatment knowledge

This theme captures accounts that describe the knowledge, sources of information and attitudes participants had prior to receiving ECT.

For many participants, ECT was perceived as an outdated method of which they had little knowledge before undergoing it themselves. Many participants had associations from movies, a musical (‘Next to Normal’), media or figurative art, which were often dramatic ECT representations. When the clinicians proposed ECT as a treatment, some participants were motivated to try it, as they had not responded to other treatments. For many of the participants, however, the first reaction was fear or hesitation. As one participant noted:

*“I remember the first time the psychiatrist in the private health service proposed it, and I totally—I completely freaked out at the thought”. (P.2)*

One next-of-kin present in the interview and two participants were health professionals and had more updated information. Two younger participants, who were not hospitalized when ECT was proposed, discussed the choice of ECT with their clinician thoroughly and actively sought information. One participant mentioned that she watched a YouTube video: “*I was just curious how it was going to be because you’ve only seen it on TV, right? And it looks different, and I had seen some videos somewhere, and there they show exactly how it is, and it’s not so bad”. (P.20)* The other younger participant referred to more contemporary and public personalities that had received ECT, such as actress Carrie Fisher or writer Ernest Hemingway. This participant noted that he was aware of Hemingway’s negative account of his experience, but he was determined to look beyond the often-negative representations of ECT and associated risks in popular culture, so he turned to academic articles for information instead.

### The experience of informed consent

This theme brings together the experience of consent from information received in the clinic, perceptions of decisional capacity and support in decision-making.

#### Information provision

Participants’ experience with information received in the clinic varied. Some participants thought the information they received before treatment was sufficient—they did not have unanswered questions, and felt involved in the decision. Others were more hesitant, needed more information, and time to think and discuss. Many did not recall signing an informed consent form but recalled that they were presented with some information explaining how modern ECT was different from earlier ECT approaches. Participants also described experiencing care from nurses and anaesthetists, who reduced their anxiety by explaining procedures.

Many participants reported that their consent was based on short conversations with the psychiatrists, nurses or other patients, in the absence of written or more patient-friendly information: *“I got a bit unsure after I signed the consent about what treatment implies. Then a nurse came with a folder with some information”. (P. 9)* One patient in her teens learned about ECT from her co-patients, many of whom had been diagnosed with schizophrenia. She also mentioned that she was not inclined at the time to think about long-term consequences: “*I had had anaesthesia before and I thought it was exciting. And that moment before you fall asleep—that thrill was very attractive. I was a bit there at that level, so I did not think through at all. (P. 5)* Participants had different needs for information and many reported experiencing reduced capacity and motivation to process information and make decisions: “*I can’t think how they could have informed me differently, because no matter how they would have formulated it, I wouldn’t have felt capable of consenting”. (P.10)* Some participants reported that presentation of ECT as a last resort made them more passively accept the decision. Five participants had been hospitalized under the Mental Health Act and although they did not receive involuntary ECT, they recall that this reduced their assertiveness.

#### Support in decision-making

Many participants also reported that information delivery was not enough, as they lacked engagement and support in decision-making from health professionals. One participant, a health professional herself, had a positive experience with ECT, and pondered whether she could have been more actively involved in the treatment. She describes how she had refused ECT in her first depressive episode. In the second episode, her physician suggested ECT much more rapidly. She was also more motivated to agree on ECT this time as she found her emotional pain unbearable even if only for some weeks.

*“I was a calm patient so I don’t understand why they did not bring [ECT] up to me, but I had some paranoid thoughts and it isn’t easy to make a patient take their medication like that (…). I wish they would have worked more with me to make me understand that I’m not locked in an acute ward, [that] they don’t want to hurt me. [I wish they had] talked to me, but it’s easy to say that in retrospect”. (P. 21)*

Many mentioned the importance of repeated discussions, or being provided written material with more information, at different time points. “*I believe talking is better than a leaflet on depression. When you’re depressed, you can’t really take it in, there has to be somebody who sits down with you and repeats and repeats that same thing many times”. (P.21).*

Participants who had prior experience with ECT noted that this made them more assertive at the next treatment: “*After the previous hospitalization, they knew me a bit—I was part of the decision. So the doctor asked me, ‘So what do you think you need?’ And I said ‘ECT has helped me before’. Then she went, ‘Okay, so we’ll do it this way. (P.10).*

Some participants in this study mentioned that they discussed the decision with family members but they would have liked to talk to or listen to others’ experiences of how they dealt with their thoughts or fear. This would have helped them ask questions about how ECT ‘feels’ and assist their decision-making.

#### Information on adverse effects

Almost all participants were anxious about memory loss, and reported that they received information that adverse effects, especially regarding memory, would be short-term and reversible. Some, but not all, received information about the risk for longer-term effects.

*I don’t remember the information given in 2011, but in 2013 I remember ECT was mentioned as a possibility because I had problems with medication. But in 2013, I remember I specifically said that I was worried my memory would be affected. And the clinician took it seriously but managed to convince me that it would only be for a short period of time, and all the evidence says that—that memory comes back but that it varies. I wonder whether this variation has to do with the number of treatments. (P.19)*

The participants who experienced antidepressant effects of ECT without adverse effects were more likely to report that they were cognizant of ECT’s adverse effects, but less aware or convinced of beneficent effects.

### The need for in-depth information on ECT

This theme brings together participants’ claims about the need for varied information throughout the treatment period.

#### Available general information

Participants continued to try to make sense of ECT throughout the treatment period, and they expressed having an overall lack of knowledge during this time. They were interested in information about specific aspects of treatment, including statistics, brain science and others’ experiences. “*I think that people missing more specific research and publishing around the results is a recurring topic—there isn’t even a national registry. (…) I always have an underlying feeling that ECT isn’t researched enough”. (P. 19).*

#### Present ECT earlier

The participants who had experienced antidepressant effects without long-term cognitive adverse effects reflected on whether ECT could have been presented as an earlier option. Participants proposed that both health professionals (e.g., GPs) and society in general should be updated with knowledge about ECT’s antidepressant effects to balance the information on risks: “*That there’s a treatment that can actually really save you (…), because I wasn’t really aware of it (…), and I’ve experienced a lot of adverse effects with medication. (P. 19).*

#### Brain health

Other participants had several ECT series, and did not recall that they could discuss the different concerns about memory or brain throughout the treatment period: “*What was completely missing was information on the long-term perspective (…) because one thing is the first time, or the second, but for me who’s had almost 10 rounds, what happens then? It hasn’t been a topic.”(P.2)* One participant expressed the need for more information on brain health and self-care after what she felt was a very intrusive intervention: “*Another thing I wish I’d had is information that the brain is vulnerable after having had ECT, so even if you feel normal it’s still important to have a calmer lifestyle—yes, and reduce [your] activity level when you come home (…). I felt that my brain was vulnerable and I got back a feeling of sadness and got easily tired when I came back home”. (P. 21)* In particular, the participants who underwent repeated ECTs expressed their worries, and a need for information, about the treatment’s effect on the brain. One of the participants—a woman in her 60s—described how her fear of dementia could lead to her underreporting of memory problems, and she wished this aspect was addressed in her conversation with the clinical team. She also gave herself quizzes to assure herself that her memory was not deteriorating.

#### Encountering different attitudes

The general lack of information, together with encountering varying attitudes among health professionals, gave participants an understanding of ECT as a less-scientific and second-best treatment. One participants noted: “*I think that it’s important for those who are for this treatment—it’s almost like they have to defend it (…) and I’m thinking they don’t need to. They are experts— [they should] put it forth as neutrally as possible … Now I feel that it gets very difficult for the patient, that you experience scepticism on the one side, from psychologists that can say ‘I don’t believe in it’, and all of a sudden you’re hospitalized in an acute ward, which strongly supports ECT. (P. 19)*

### Life after ECT

This theme brings together descriptions of adverse effects and ways participants dealt with these experiences.

#### Post ECT expected amnesia

Seven participants reported that they only experienced amnesia for the period before and after ECT, which they had been informed about it. Two of these experienced some reduced memory function, such as struggles to remember text when reading. They were satisfied with the ECT overall, and they were not certain whether to attribute these struggles to ECT. A few participants told of neuropsychological monitoring tests during ECT and a few treatment sessions were stopped due to confusion. For some participants, even the short-term temporary confusion and lack of memory around everyday knowledge represented experiences that had a strong impact on their sense of self: *“what I reacted on was that they say it’s only trivial things you forget but I forgot much more”* (P. 9).

#### More memory struggle than expected

Eight participants experienced more memory struggles than described in the consent information. These participants describe dysfunctions like struggling to remember names and appointments, less concentration or a slower processing speed. They conveyed that, though they were able to regain some of their memories or knowledge, and some were able to study, it felt like a greater struggle to remember: “*It’s okay with remembering—I remember/memorize normally, it’s just that I don’t remember like I used to. It’s different”. (P.10)* Another participant told: “*But that I don’t remember that wedding—I was hospitalized at that time, and because usually you talk about things, and it takes a few years to find out, and I know where it was, just that I wouldn’t know if nobody ever asked me (…) or if I didn’t have pictures of it”. (P. 5).*

Several participants reported that the general feeling of inferior cognitive performance after ECT sometimes affected their social life and self-confidence, although it appeared tolerable for some: “*Now I can almost start doubting what someone else said: Did they say it or not, how was it actually? Before, I was stubborn, I used to be, but now I can’t be that stubborn because I don’t have control of what I remember or not. But I think it’s also a matter of practicing”. (P.20).*

#### When costs are higher than benefits

Six participants held more strongly critical views towards ECT given the costs to memory, but also lack of adequate information or acknowledgement. They discovered that they still had cognitive struggles some time following the treatment, in social and work-related contexts; they noted that these contexts triggered their lack of ability to recall events or knowledge. One participant described how it took some time to discover these difficulties: “*It took a few years before I was aware because I wasn’t very attentive to it, and I would’ve discovered it faster if it would’ve been today. But then I wasn’t very interested in remembering things—I didn’t need my brain. I was in high school at that time, and I took my exams and I was doing fine” (P.5).* Some participants spoke of ‘holes’ in their personal memories, and the experience of unrecoverable memories had a strong impact on their sense of self. Especially one participant described that severe loss of memory made her feel she lost herself: “*it’s like I have not existed before”* (P. 14). A few others experienced that they also lost knowledge related to their work life that they could not recover.

#### Coping strategies

The participants who experienced memory struggles told of strategies they employed to reconstruct their autobiographical memory, like looking at photo albums, talking to friends and family, and *‘doing the work of tying together loose threads, so I spend some time on reading [medical] logs and asking family what they remember’ (P.11)*. One participant described how she had to relearn factual, work-related knowledge: *‘My house was full of Post-it Notes’ (P. 3)*. Others reported strategies that they used to support memory in their everyday life, such as writing down appointments immediately, taking notes or repeated reading.

#### Lack of support

While in treatment, only one participant told of a nurse who kept a log for her during the treatment time: “*I don’t remember very much from that time period but my responsible nurse wrote a diary which is worth gold now”. (P. 7) P*articipants pointed to a lack of available information that could help them understand and cope with their experience of coping with adverse effects after discharge. They conveyed that they would have liked to know about others’ experiences in order to understand their own. While some participants found others to talk to, or read about others’ stories with ECT, overall, they reported scant access to reliable sources. Many participants acknowledged that discerning the underlying cause of memory problems is a complex issue, and that it is challenging to distinguish the effects of ECT from those of illness or medication. They also described a disconnect between their experiences and professionals’ understanding, as the latter often attributed memory complaints to depression and were less willing to address participants’ concerns about cognitive functioning: *‘I feel that those that inform are not up-to-date about how much it actually is [memory loss]’ (P.1).*

One participant who experienced side effects, which she found troubling, was referred further to the neuropsychologist by her treating psychiatrist, although this is not standard practice. However, she reported that the session with the neuropsychologist did not bring more understanding, comfort or support. Instead, her psychologist addressed some of her concerns:

*“Like my psychologist said, ‘You still have the ability to learn’. So luckily I haven’t gotten Alzheimer’s, but I have to repeat and repeat and it isn’t enough that I go that route one more time”. (P. 19)* Some accepted the cost and felt they could live their life despite these limitations. Others were more resentful of the lack of information, support and acknowledgment.

## Discussion

This study explored ECT recipients’ experience with treatment information, and revealed that limited knowledge, negative perceptions and/or fear of memory loss made participants hesitant about ECT, but did not deter them from consenting to it. This finding is consistent with that of other studies [22]. Study participants also highlighted a lack of sufficient information provision in and outside the clinical setting. This is concerning, considering that participants have reported the clinic as their main source of information, and scarce or biased information as coming from other sources or society [34, 35].

Health professionals may have been focused on motivating and reducing patient fears about ECT, and a majority of the participants reported being sufficiently informed about the procedures in this respect and feeling taken care of. Other participants described an informed consent process that was often based on brief conversations with the health care team, a lack of varied and thorough information, and scant support in decision-making. Several participants questioned the validity of their consent, as they were unable to process the information, lacked knowledge or felt they had no alternative. Participants emphasized the need to have better access to balanced, updated and adequate information through different modalities and at different times throughout the treatment. Having available, updated and reader-friendly material that can be discussed repeatedly and openly with health professionals could help participants make informed decisions; indeed, other studies have proposed and reported the use of video in this context [23, 26].

While the passivity that some participants described may be seen as reflecting trust in health professionals, it could also indicate a lack of engagement—a finding confirmed by other studies [22, 36]. A more careful description of ECT than simply ‘a last resort’ treatment may help encourage active patient engagement in treatment decisions, and may adjust their expectations and prevent disappointment [35]. One study has shown that even standard patient education (via discussions and video) could improve understanding and decisional capacity for ECT recipients. Interestingly, however, the study found that an additional intervention of an extra half hour with a psychiatrist did not result in measurable improved decisional capacity—indicating a challenge around determining the appropriate amount of information for optimal decisional capacity [26]. In the present study, participants conveyed the need for information in relation to consent but also throughout the treatment process. Several studies have underlined the role of both the psychiatrist and the nurses to provide information and support in decision-making. The latter’s therapeutic relationship and presence in patients’ everyday life allows for good explorative conversations [36, 37]. Wells et al. suggest that support in decision-making can also come from peer workers, as this information may be perceived as ‘more balanced’ [23]; the authors propose that peer workers could provide descriptions of procedures while individuals ‘wait in line’, to reduce anxiety or follow-up with participants’ need for information. A few participants in the present study saw a role for peer workers in the consent and decision-making process, although they were not aware whether this already existed; here, they problematized the importance of ‘balance’, given that others’ ECT experiences could be either positive or negative. Further, participants saw a role for peer workers during and after treatment, so that they could help recipients discuss, understand or cope with their experiences—with amnesia being the biggest concern.

The participants who experienced an antidepressant effect without adverse effects, or who felt that the benefits outweighed the costs, pointed to a need for more positive narratives and updated research on ECT. They suggested that presenting ECT as an evidence-based method—and not only as a last resort—both to health professionals and the public may help normalize and de-stigmatize the treatment: a finding that also resonates with the findings and suggestions of Wells et al. [35]. In contrast, participants who experienced some form of amnesia reported how the information on adverse effects they received in the consent process was minimalized. Some participants held negative expectations of ECT, which may explain their persistent subjective complaints [15], while others had on the contrary high expectations from ECT. Participants expressed a willingness to accept some treatment costs, as they engaged in cost–benefit analysis, but they found it difficult to assess how more comprehensive risk information would influence their decisions. In order to help recipients feel adequately informed and cope with amnesic experiences, active use of diary writing, documentation of information given, and patient and family psychoeducation have been suggested [14, 22, 23]. Moreover, this ethical dilemma of adequate risk information highlights the importance of the clinician’s skills in communicating and acknowledging the changes that can occur, not just as abstract probabilities but also as changes in the ECT recipient’s experience of the self. For example, difficulties reading books, seeing movies and recognizing faces or names—as the present study and others report—are not simply attentional problems, but problems of ‘seeing’ the world as a whole [38]. The present study supports the assumption that the mechanistic approach to information, as opposed to one that is phenomenological inspired, fails to prepare and support individuals when dealing with potential adverse effects of ECT. This approach’s limitation might also explain the continued reports of dissatisfaction with information about adverse effects, even though individual and contextual factors might explain that dissatisfaction [39, 40].

This unpreparedness and lack of support in coping with memory complaints might in fact reflect the knowledge gaps and limited efforts to investigate remediation therapies and methods to reduce adverse effects other than the different ECT technical methods [24, 41]. The current batteries of tests still do not measure all aspects of subjective memory complaints, and there is no consensus about cognitive domains, appropriate tests that can predict daily function, or the tests’ timing [16, 41]. Those participants who reported memory impairments employed various strategies to remember or reconstruct knowledge, but experienced a lack of monitoring services and professional interest after discharge. Wells et al.’s findings suggest that researchers’ focus on suffering has hindered systematizing knowledge about coping strategies after ECT; these gaps hinder health care services from monitoring and supporting individuals coping with adverse effects, allaying fears or correcting misattribution about potential memory loss due to ECT [42].

Study participants also experienced a lack of factual and narrative information that could increase their understanding and coping, and reduce their feelings of stigma for receiving a marginalized treatment [34, 35]. ECT is perceived as more stigmatized than other treatment methods as it is associated with unwanted characteristics (e.g., personality change and brain damage) [35]. Thus, experiencing adverse effects and gaps in knowledge and services for an already stigmatized treatment may lead to individuals internalizing negative self-evaluations of themselves as ‘different’ or ‘abnormal’.

### Strengths and limitations

This is the first Norwegian study, which also includes user involvement, to map participant experiences with ECT in a young sample. The researcher had a neutral stance however some limitations may skew the study’s findings, specifically concerning the challenges around finding a balance of positive and negative voices—even if participants are recruited through university sites [22] or a large mental health organization. However, the participants’ accounts were complex, and not simply ‘for’ or ‘against’. A few participants in this group, not recruited through hospitals, participated in the study to voice their positive stories in a context where ECT’s (re)presentation is predominantly negative. The negative accounts of some participants may also be explained by the older treatment methods (eight participants received ECT between 1999/2000–2010), or by the longer time interval since their last ECT, which tends to predict more dissatisfaction [22]. Memory loss inherent to the treatment can also explain some of the reported lack of information; however, memory loss commonly reported after ECT is an unavoidable aspect of researching patient views and should not automatically discredit participants’ accounts [19]. A final limitation is that this study did not systematically analyse the informed consent information from each institution.

## Conclusion

Despite the limitations identified, the study supports the findings of other studies and calls to increase patients’ access to varied facts and stories, in the clinic and on communication platforms, as well as support patients coping with potential adverse effects. These efforts could bring ECT services in line with a more patient-centred care model [23, 43, 44] and reduce the procedural injustice [36] and structural discrimination [35] of a subgroup of individuals. Further research should monitor how enhanced patient education influences consent processes, satisfaction with information and treatment.

## Data Availability

The datasets generated and analyzed during the current study are not publicly available due to data protection regulations but are available from the corresponding author on reasonable request.
